# Evaluation of saps-3 index in patients whith structural brain lesion admitted in intensive care unit (ICU)

**DOI:** 10.1186/2197-425X-3-S1-A833

**Published:** 2015-10-01

**Authors:** C Lopez-Caler, MD Arias-Verdú, R Gónzalez-Ortiz, J Moreno-Quintana, E Castillo-Lorente, E Aguilar-Alonso, J Barrueco-Fanconi, G Jiménez-Pérez

**Affiliations:** Intensive Care Unit, Hospital Regional Universitario Carlos Haya, Málaga, Spain; Intensive Care Unit, Hospital Neurotraumatologico, Jaen, Spain; Hospital Infanta Margarita Cabra, Córfdoba, Spain; Intensivo Care Unit, Hospital Regional Universitario Carlos Haya, Málaga, Spain

## Objectives

To evaluate SAPS 3 performance in patients with structural brain pathology (traumatic brain injury (TBI) and acute cerebrobrovascular accident) admitted in ICU.

## Methods

We studied all patients admitted with structural brain pathology (traumatic brain injury and acute cerebrobrovascular accident) in three Spanish Hospitals (Hospital Carlos Haya de Malaga, Hospital de Cabra and Neurotraumatológico de Jaén) during four months in 2012 and 2013. We collected clinical and demographics data, mortality and the necessary data to calculate the SAPS-3 indexThe differences between observed-to-predicted mortality were analyzed with the Hosmer-Lemeshow test. SAPS 3 discrimination with regard to hospital mortality, tested using the area under the ROC curve.

P < 0.05 was statistically significant (s.s).

## Results

N= 128 patients. Traumatic brain injury: 53 patients. Stroke: 75 patients. Mean age was 56.23 ± 18.56 years. Gravity according to SAPS-3 was 51.69 ± 18.56 points. Mortality predicted by the SAPS-3 was 25.57% by the general equation and 26.12% by geographical area equation. Hospital mortality was 31.2%.We divided the population according to predicted mortality by the general equation of SAPS-3 in: 1) less than 20%, 2) between 20-40%, 3) 4 0-60%, 4) 60-80%, and 5) greater than 80%. The predicted mortality was respectively: 8.2%, 28.3 % 49.2%, 68.4% and 84.8% and the observed was 6.2%, 30%, 75%, 91% and 100%; H = 8.84, GL = 3, being the differences between predicted and observed statistically significant (p < 0.05). By the geographical area equation we found similar disagreements; H = 10.61 (p < 0.05) SAPS 3 discrimination with regard to hospital mortality, tested using the area under ROC curve was high: 0.90 (0845-0961).

## Conclusions

Patients with structural brain lesion admitted in ICU (TBI and stroke) have a high value of severity assessed by SAPS-3. The SAPS-3 presents good discrimination in these patients but slightly underestimates mortality, being no very important differences between predicted and observed mortality, but statistically significant.

